# Blood Donation Knowledge, Perceptions, and Practices during COVID-19 Pandemic: Questionnaire-Based Study in Saudi Arabia

**DOI:** 10.1155/2023/3911907

**Published:** 2023-04-12

**Authors:** Hisham Ali Waggiallah

**Affiliations:** Department of Medical Laboratory Sciences, College of Applied Medical Sciences in Alkharj, Prince Sattam Bin Abdulaziz University, Alkharj 11942, Saudi Arabia

## Abstract

**Results:**

The level of good knowledge of the blood donation process and perceptions constituted more than 52.2% and 52.9%, respectively, of the participants. The biggest motivation for the donation process was the awareness campaigns, which amounted to 52.5%, and the biggest obstacle to the blood donation procedure is the lack of health fitness. It has been found that 43.0% of those who completed the questionnaire had donated blood during the COVID-19 pandemic.

**Conclusion:**

Though the high level of awareness of the importance of blood donation for COVID-19 patients, as well as the satisfaction with the experience of blood donation for patients, the level of general knowledge remains average.

## 1. Introduction

Blood transfusion is an important service for health systems around the world, which suffer on a daily basis from having to make a balance between population requirements and the amount of donated blood provided. Before the COVID-19 pandemic, developed-country healthcare systems were already observing an alarming reduction in blood donations [1–3], which was worsened by lockdown, social isolation, and cancellation of blood collections, as well as additional exclusion criteria [4–6]. More obstacles have emerged for the blood transfusion community, on top of lockdown, social isolation, and fear of blood virus transmission. The senior group, which gives the most in donations, is among the most vulnerable to the virus, resulting in lower donations from them [7]. Moreover, the number of asymptomatic cases increased, making it more difficult to diagnose and, as a result, posing an indirect danger to blood collection safety [8, 9]. As a result of decreasing donations, hospitals were obliged to conserve supplies by canceling nonurgent procedures and organ transplants or limiting transfusion volumes in order to avoid blood insufficiencies [10, 11]. Though reducing the need for blood initially worked, an increase in blood component wastage was also found as a result of the decrease in demand matching the blood products' limited life span [11]. The idea of moving blood products between different blood collection centers was similarly unfeasible. During the initial severe acute respiratory syndrome coronavirus 2 (SARS-CoV-2) outbreak, China attempted to do so but encountered temperature and packing issues, resulting in increased errors, expenses, and waste [12, 13]. Saudi Arabia has made considerable strides toward providing safe blood for its citizens. Over the last three decades, the blood supply has changed from imported blood to local voluntary donors [14]. This has been made possible by the highly efficient blood transfusion service (BTS) as well as outreach operations to prospective donors [15]. The goal of this study is to assess blood donation knowledge, perceptions, and conceptions during the COVID-19 pandemic.

## 2. Material and Methods

### 2.1. Study Design and Setting

A cross-sectional study was conducted during the period between March 2021 and September 2021 on Saudi population from 13 administrative rejoins.

### 2.2. Study Population and Sampling

Online survey was used to reach out to the various population categories. The total sample size for the current study was 314 participants.

### 2.3. Data Collection

The questionnaire was composed of 4 parts. Part one: sociodemographic characteristics, like age, occupation, education level, residence, marital status, college, and discipline. Part 2 focused on the participant's knowledge about blood donation. The participant's knowledge was assessed by nine questions where zero point was given for an increscent answer and one point was given for a correct answer. The maximum possible score was seven points with a range from zero to twelve points.

The perception was assessed for blood donation by using 8 questions; some of them are yes/no questions and others with multiple responses. Participants were given one point for each answer that reflects a positive attitude and zero points for each answer that showed a negative attitude. The maximum possible score was 11 points with a range from zero to 11 points. Part 4 focuses on practices. The practices were assessed through three questions. Participants were given one point for each activity related to blood donation and zero point for not practicing any activity related to blood donation. The maximum possible score was five points with a range from zero to five points. To ensure the clarity and relevance of the questions and to determine the time required to answer all items, the questionnaire was validated by public health physicians and specialists. Moreover, the questionnaire was pilot tested among a simple random sample to check its format, language, sequence, comprehension of the questions, and duration. Cronbach's alpha, which was (0.72) for knowledge, (0.71) for perceptions, and (0.70) for practice, was used to assess the tool's reliability and validity (0.77); the confidentiality and privacy of data were maintained.

### 2.4. Data Analysis

The Statistical Package for Social Science (SPSS) version 24 was used to analyze the data. Descriptive statistics were used to calculate frequencies and percentages for categorical variables and obtain mean and SD for continuous variables which are presented in tables and graphs. The scores of the knowledge, attitudes, and practices were dichotomized by using the mean as cutoff points. When the score of the knowledge was equal to and greater than the mean score (10.4 points), the participants were considered to have good knowledge while less than the considered had a poor level of knowledge about blood donation. Also, regarding the attitude, if the score of the participants is equal to or greater than the mean score (9.2 points), they were considered to have a positive attitude. In case of practice, the same strategy was followed if the participants had practice scores equal to or more than the mean considered hand good level of practice and vice versa. Logistic regression analyses were used to identify factors associated with blood donation behavior adjusted by confounding factors like age, gender, marital status, education, and discipline. The tests were carried out at 95% confidence interval, *p* values less than 0.05 were considered significant, and odds ratio (OR) with 95% confidence interval (CI) was used to assess the presence and degree of association between dependent and independent variables. A two-sided *p* value < 0.05 was considered statistically significant.

### 2.5. Ethical Approval

Before the study began, ethical approval was obtained from the Research Ethics Committee, College of Applied Medical Science, Prince Sattam Bin Abdulaziz University.

## 3. Results

### 3.1. Sociodemographic Characteristics

Table 1 contains social and demographic data, such as the average age of those who participated in the study, which was between 20 and 24 years old, accounting for approximately 41.4% of those who participated, and also, it has been revealed that males made up the majority of those who participated in the study, accounting for 80.3% of those who participated. The percentage of people who took part is 69.4%. At the educational level, bachelor's degree holders made up the largest group in the study, accounting for 62.4% of the total, which is equal to the percentage of students in the study. The majority of participants in the study came from cities, accounting for approximately 96.5% of those who took part.

### 3.2. Health Status of Study Participants

The study's participants' health state ranged from the highest percentage of smokers, 21.0%, to the lowest percentage of metabolic syndromes, 19.7%. Hematological, immunological, and infectious diseases comprised 5.7%, 3.5%, and 1.0% of the total, respectively; the rest of the respondents were considered healthy (49.1%) as displayed in Table 2.

The ability to accurately answer the majority of knowledge questions is defined as having good knowledge, perception, and practice. The majority of knowledge questions are answered incorrectly due to a lack of knowledge, perception, and practice [16]. Respondents were asked to complete a series of questions designed to assess their awareness and understanding of blood donation, as well as their attitudes and factors that hinder or encourage them to donate as shown in Tables 3–5.

### 3.3. Knowledge

Table 3 shows the participants' knowledge of the relevance of blood donation in the COVID-19 pandemic, and it has been discovered that the general level of good knowledge was 52.2%, while the bad knowledge was 47.8%.

Poor knowledge was associated with questions about the appropriate age for donating blood, conditions that prevent blood donation, and the probability of donation. Before donating blood, a donor's hidden disorders can be uncovered. Otherwise, the knowledge was adequate for the population who took part in the questionnaire.

### 3.4. Perceptions

Table 4 shows the perception of the benefit of blood donation, which reveals a general level of positive perception of blood donation of 52.9%. The perception of procedures inside hospitals for donating blood during the COVID-19 pandemic was higher than average and not completely satisfied at 78.7%, as were responses to questions about future blood donation campaigns, which were 65.9% positive, and the reexperience of blood donation, which was 70.1% positive.

### 3.5. Practices

Table 5 describes blood donation practices during the COVID-19 pandemic. It was found that the overall positive practice of blood donation was only 43.5%. It was revealed that 43.0% of those who responded to the survey had donated blood.  Table 6 lists the factors that encourage and discourage research participants from donating blood. It was noticed that the greatest motivator for the donation process, accounting for 52.5%, is a lack of health fitness and that the greatest impediment to the blood donation procedure is a lack of health fitness.

**Figure 1:**
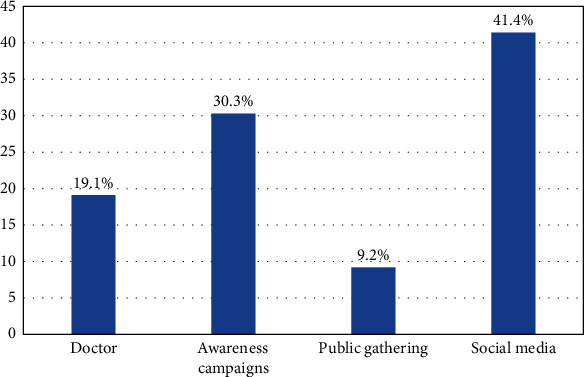
The source of knowledge about blood donation (*N* = 314).

Figure 1 depicts the source of awareness regarding blood donation, which is led by social networking sites (41.4%).

Multiple logistic regression models adjust for age, gender, marital status, and discipline. The study results showed that participants who had good knowledge about blood donation (OR, 1.916; 95% CI, 1.168–3.144) were more likely to donate blood than those who had poor knowledge of blood donation. In addition, the results showed that age and gender covariates indicated a positive, significant association with blood donation. Specifically, the age group (20–24 years) was found to be 2.5 times more likely to donate blood (OR = 32.523, 95% CI = (1.786–2.954), *p* ≥ 05), while males were more likely to donate blood than females (OR = 8.30, 95% CI = (3.352–20.549), *p* ≤ 0.05). However, marital status and discipline were not associated with blood donation. This means each category has a similar probability of donating blood as mentioned in (Table 7).

Several logistic regression models are used to account for age, gender, marital status, and discipline. Participants who had a positive perception of blood donation (OR = 0.426, 95% CI = (0.260 − 0.698), *p* ≤ 0.05) were more likely to donate blood than those who had a negative perception of blood donation. Furthermore, the findings revealed that age and gender variables had a favorable, statistically significant relationship with blood donation. The age group (20–24 years) was shown to be 2.5 times more likely to donate blood (OR = 2.432, 95% CI = (1.735 − 2.790), *p* ≤ 0.05), while males were more likely than females to donate blood (OR = 8.527, 95% CI = (3.428 − 21.214), *p* ≤ 0.05). However, marital status and discipline had no effect on blood donation. This means that each category has a similar chance of donating blood as shown in Table 8.

## 4. Discussion

The current study presents awareness, perception, and concepts related to blood donation during the COVID-19 pandemic in Saudi Arabian society, as well as other factors such as information about the motivators, barriers, and communication channels related to blood donation among Saudi society segments, as well as data that can help blood transfusion centers design targeted programs to encourage donors and work to increase their number.

Young individuals aged 20-34 years, who account for slightly less than half of all participants, were the most actively involved group in this study, accounting for 41.4% of all participants. In the previously mentioned Riyadh study [15], it was revealed that the age group 15-30 years was the least likely to donate blood. The reason that this age group predominated in all studies due young individuals is more motivated, enthusiastic, and healthier than other age groups.

In terms of educational level, it has been found that bachelor's degree holders have the greatest involvement rates, and participation rates are equal among students and nonstudents. The majority of those who took part in this study live in cities. In terms of participants' health, the percentages of smokers and those with metabolic diseases converged. The result of the current study is relatively inconsistent with the previous study revealed that approximately 71% of those polled said they had attended a university. Because this survey was conducted in city stores, clinics, and via social media, the vast majority of participants are anticipated to be urbanites with a higher educational level and greater exposure to health communication materials than populations residing in the countryside or those who do not use social media or the internet to some extent [17].

In the present study, knowledge status of blood donation during the COVID-19 pandemic, more than half of the participants (52.2%) (*p* value = 0.01) with good knowledge of the blood donation process accounted for it. Our finding is, to some extent, similar with the previous study; most of the participants were more inclined to donate blood (*p* = 0.001). This is significant since in previous studies, those who did not donate blood claimed a lack of awareness of the necessity for blood donation as a reason for not doing so. According to the Riyadh study [15], 69.5% of those who had never given before were unaware that blood banks needed blood. Similarly, in another study [18], almost 435 individuals said that they had not been solicited to donate blood because they had not been approached by anyone. The level of knowledge can be raised by instilling a culture of blood donation among individuals and organizing camps and field trips in public places such as parks, shopping malls, and universities. Furthermore, the media, in all of its forms, should be capable of emphasizing the importance of this issue and emphasizing the need for volunteers to cover the lack of blood donation. People can donate blood more easily, thanks to mobile blood-collecting vehicles [19, 20].

In this study, it was observed that 43% of participants donated blood during the COVID-19 pandemic, whereas in a previous study conducted in the Asir region, south of the Kingdom of Saudi Arabia, the percentage of blood donation prior to the pandemic was 35% 16, and other studies in Ethiopia (35.0%) [21] and Jordan (29.0%) [22]. Another old study 15 conducted in Riyadh among the general population in 2005–2006 indicated a blood donation prevalence of roughly 34%, while another study [23] conducted among students in Jeddah in 2013 found that only about 20% of the individuals had previously donated blood. Another study [24] conducted among health professions students in Jeddah in 2014–2015 found that approximately 30% of those polled had donated blood. All these previous studies indicate that there is an increased awareness of the importance of blood donation during the COVID-19 pandemic, which is a result of raising awareness through social media sites, which constitute 41% of information sources for Saudis.

In this current study, it has been discovered that positive perceptions during the COVID-19 pandemic comprised 52.9%, the percentage of satisfaction with blood donation was a very high rate of 98.4%, and the feeling of the importance of donating blood for COVID-19 patients was also a very high rate of 99.4%, while 93.0% of participants expressed a desire to transfer their positive experience of donating blood to others. It was observed that awareness campaigns, which stimulated a rate of 52.5%, were one of the biggest motivators for donating blood during the COVID-19 pandemic. A previous study revealed that the most prevalent two reasons cited as impediments to blood donation were health status and a lack of opportunity or time. However, because the majority of the participants did not mention their health issues, it is impossible to establish that all of these health issues are true barriers to blood donation [25].

Other research displayed important motivators such as “solidarity” or the “satisfaction of helping others” [26]. Previous studies found substantial differences in motivators such as “a family member or friend who needed a transfusion,” “family members or friends who are donors,” and “information or promotion campaigns” [27]. Donors and key informants indicated altruism, or they want to do something great for somebody without getting anything in return, as the top motivator for giving blood [28]. Despite increased awareness of the importance of blood donation, the COVID-19 pandemic exacerbated the blood donation process through lockdown, social isolation, and cancellation of blood collection processes, as well as concern about blood virus infection. However, because the government focused on everything related to the COVID-19 pandemic by increasing awareness campaigns, knowledge, attitude, and practice toward the blood donation process increased.

## 5. Limitations

In the present study, the main barrier to the blood donation procedure was the blood donor's lack of fitness, which constituted 35.7% of all obstacles faced. Individual impediments to blood donation include feeling unwell, a lack of time, fear of the donation process or transmission of infection, a lack of proper education, an edge, and a lack of trust in institutions. The most prevalent two reasons cited as impediments to blood donation were health status and a lack of time. However, because the majority of participants did not mention their health issues, it is impossible to establish that all of these health issues are a true barrier to blood donation.

## 6. Recommendations

Facilitate access to blood donations near major workplaces and improve awareness and the effectiveness of the service to recruit individuals by intensifying blood donation propaganda campaigns and focusing on social media, which emerged as the primary source of information during the COVID-19 pandemic, as well as urging medical staff to spread the culture of blood donation and developing other means to raise awareness of the importance of blood donation as a basic strategy for health services in the Kingdom of Saudi Arabia.

## 7. Conclusion

This is the first study to determine the level of awareness of the blood donation process during the COVID-19 pandemic. In spite of the dilemma posed by COVID-19 at the health sector level, it has been revealed that knowledge, attitude, and practice of blood donation have increased. This is due to the tremendous efforts made by the health services during COVID-19, but it is necessary to raise awareness to a greater degree about donating blood in general as a vital and strategic service for the health sector in the Kingdom of Saudi Arabia.

## Figures and Tables

**Table 1:** Sociodemographic characteristics of the study respondents (*N* = 314).

Variables	No.	%
*Age group*		
(i) 15-19	58	18.5
(ii) 20-24	130	41.4
(iii) 25-29	30	9.6
(iv) >30	96	30.6
*Gender*		
(i) Female	62	19.7
(ii) Male	252	80.3
Marital		
(i) Married	90	28.7
(ii) Single	218	69.4
(iii) Divorced	6	1.9
*Education*		
(i) Intermediate and lower	4	1.3
(ii) High	71	22.6
(iii) Bachelor	196	62.4
(iv) Postgraduate	43	13.7
*Occupation*		
(i) Student	196	62.4
(ii) Governmental	49	15.6
(iii) Private	37	11.8
(iv) Housewife	19	6.1
(v) Unemployed	13	4.1
*Residence*		
(i) Urban	303	96.5
(ii) Rural	11	3.5

Mean age of the respondents = (28.0 ± 11.7) years.

**Table 2:** Health status of study participants (*N* = 314).

Variables	Responses		
	Yes	No	95%, CI^∗^
	(no./%)	(no./%)	
(i) Smoking status	66 (21.0)	248(79.0)	16.5-25.5
(ii) Hematological disease	18(5.7)	296(94.3)	3.1-8.3
(iii) Any immune diseases?	11(3.5)	303(96.3)	1.5-5.5
(iv) Any metabolic syndromes?	62(19.7)	25.2(80.3)	15.3-24.1
(v) Infectious diseases	3(1.0)	311(99.0)	0.1-2.1

^∗^95%, CI: for the proportion of the participants with health condition.

**Table 3:** Knowledge of the study participants about blood donation during COVID-19 online survey, December 2021 (*N* = 314).

	Knowledge questions	Correct responses	
		No	%
1	How often can an individual donate blood (yearly/every 6 months)	201	64.0
2	Any healthy adult is able to donate	303	96.5
3	Age is a limitation on blood donation	227	72.3
4	Can people with any blood group donate blood	302	96.2
5	Blood donation is important in emergency situation	301	95.9
6	Blood can be stored in the blood bank	304	96.8
7	Blood donation has beneficial effects to the donor (agree)	302	96.2
8	You will donate blood if you receive notification of the need for blood	229	72.9
9	Blood donation activates blood circulation and blood renewal	305	97.1
10	Blood donation decreases heart and arterial diseases	233	74.2
11	Blood donation detects hidden diseases	235	74.8
	Overall level of knowledge		
	(i) Poor	150	47.8
	(ii) Good	164	52.2
	Mean knowledge score (mean ± SD): 10.4 ± 1.5; range: 5)–12 out of 14		

**Table 4:** Perceptions of the study participants toward blood donation (*N* = 314).

	Perception questions	Positive perception	
		No	%
1	What is you are feeling about the procedure in the blood bank or hospitals during COVID-19?	247	78.7
2	Do you feel blood donation is important for COVID-19 patients?	312	99.4
3	How do you feel after the blood donation experience for COVID-19 patients?	306	97.5
4	Would you participate in a blood donation campaign in the future?	207	65.9
5	Would you tell other people about your donation experience?	292	93.0
6	For whom would you prefer to donate blood?	276	87.9
7	Would you donate blood again?	220	70.1
8	Are you satisfied with blood donation for COVID-19 patients?	309	98.4
	Overall level of perception		
	(i) Negative perception	148	47.1
	(ii) Positive perception	166	52.9
	Mean perception score (mean ± SD): 9.2 ± 1.2; range: 4-44		

**Table 5:** Practice of blood donation among the study participants (*N* = 314).

	Attitude questions	Good practices	
		No	%
1	Have you ever donated blood during COVID-19 pandemic?	135	43.0

2	Have you ever donated blood during COVID-19 pandemic		
	(i) Yes, every 3 months	33	10.5
	(ii) Yes, every 6 months	69	22.0
	(iii) Yes, once a year	33	10.5

3	Did you feel any complications after donating blood	10	3.2
	Overall level of practice		
	(i) Poor practices	279	56.5
	(ii) Good practices	135	43.5
	Mean practice score (mean ± SD): 4.5 ± 0.7; range: 3-5		

**Table 6:** Motivators and barriers hindering blood donation among the study participants (*N* = 314).

Motivators and barriers	Responses	Frequency	%	95%, CI
Motivators	Health care provider	39	12.4	8.8-16.0
	One of my relatives	71	22.6	18.0-27.2
	Friends	39	12.4	8.8-16.0
	Awareness campaigns	165	52.5	47.0-58.0
	Desire to donate in the future to my relatives	39	12.4	8.8-16.0

Barriers	Unfit for blood donation	112	35.7	31.4-42.0
	Fear of catching diseases	43	13.7	9.9-17.5
	Fear of needle pain	59	18.8	14.5-23.1
	Lack of special reward	5	1.6	0.2-3.0
	Desire to donate in the future to my relatives	95	30.3	25.2-35.4

**Table 7:** Logistic regression for knowledge as factors associated with blood donation adjusted by sociodemographic (*N* = 314).

Sociodemographic characteristics	Responses	Proportion (%)	*p* value	OR	95% C.I.
Knowledge	Poor knowledge	150 (47.8)			
	Good knowledge	164 (52.2)	0.010^∗^	1.916	(1.168-3.144)

Age group	15-19 years	58 (18.5)	0.320		
	20-24 years	130 (41.4)	0.021^∗^	2.523	(1.786-2.954)
	25-29 years	30 (9.6)	0.838	1.107	(0.418-2.934)
	≥30 years	96 (30.6)	0.588	0.787	(0.331-1.871)

Gender	Female	62 (19.7)			
	Male	252 (80.3)	0.000^∗^	8.300	(3.352-20.549)

Marital status	Married	88 (28.0)			
	Single	226 (72.0)	0.884	0.947	(0.452-1.981)

Discipline	Health science field	167 (53.2)			
	Other discipline	147 (46.8)	0.673	0.894	(0.531-1.505)

To identify whether knowledge associated with blood donation adjusted by sociodemographic, binary logistic regression analysis was used.					

^∗^ *p* ≤ 0.05 is significant.

**Table 8:** Logistic regressions for perceptions as factors associated with blood donation adjusted by sociodemographic (*N* = 314).

Sociodemographic characteristics	Responses	Proportion (%)	*p* value	OR	95% C.I.
Perceptions	Poor knowledge	150 (47.8)	1		
	Good knowledge	164 (52.2)	0.001^∗^	0.426	(0.260-0.698)

Age group	15-19 years	58 (18.5)	1		
	20-24 years	130 (41.4)	0.029^∗^	2.432	(1.735-2.790)
	25-29 years	30 (9.6)	0.729	1.190	(0.446-3.172)
	≥30 years	96 (30.6)	0.647	0.817	(0.344-1.939)

Gender	Female	62 (19.7)	1		
	Male	252 (80.3)	0.000^∗^	8.527	(3.428-21.214)

Marital status	Married	88 (28.0)	1		
	Single	226 (72.0)	0.955	1.021	(0.488-2.137)

Discipline	Health science field	167 (53.2)	1		
	Other discipline	147 (46.8)	0.441	0.815	(0.484-1.371)

To identify whether perceptions associated with blood donation adjusted by sociodemographic, binary logistic regression analysis was used.					

^∗^ *p* ≤ 0.05 is significant.

## Data Availability

Data is available on request.
